# Electrochemical dendrite management via voltage-controlled rearrangement

**DOI:** 10.1093/nsr/nwaf013

**Published:** 2025-01-13

**Authors:** Zhexuan Liu, Xinru Wu, Xiao Xiao, Zhiqiang Xiao, Qingjin Fu, Zhiyang Zheng, Xiongwei Zhong, Fengyi Zheng, Guangmin Zhou

**Affiliations:** Institute of Material Research, Tsinghua Shenzhen International Graduate School, Tsinghua University, Shenzhen 518055, China; Institute of Material Research, Tsinghua Shenzhen International Graduate School, Tsinghua University, Shenzhen 518055, China; Institute of Material Research, Tsinghua Shenzhen International Graduate School, Tsinghua University, Shenzhen 518055, China; Institute of Material Research, Tsinghua Shenzhen International Graduate School, Tsinghua University, Shenzhen 518055, China; Institute of Material Research, Tsinghua Shenzhen International Graduate School, Tsinghua University, Shenzhen 518055, China; Institute of Material Research, Tsinghua Shenzhen International Graduate School, Tsinghua University, Shenzhen 518055, China; Department of Materials Science and Engineering, Southern University of Science and Technology, Shenzhen 518055, China; Institute of Material Research, Tsinghua Shenzhen International Graduate School, Tsinghua University, Shenzhen 518055, China; Institute of Material Research, Tsinghua Shenzhen International Graduate School, Tsinghua University, Shenzhen 518055, China

**Keywords:** metal anode, rechargeable batteries, dendrite growth, voltage region, cycling stability

## Abstract

The pursuit of advanced energy-storage solutions has highlighted the potential of rechargeable batteries with metal anodes due to their high specific capacities and low redox potentials. However, the formation of metal dendrites remains a critical challenge, compromising both safety and operational stability. For zinc-based batteries (ZBs), traditional methods to suppress dendrite growth have shown limited success and often entail performance compromise. Here, we propose a novel strategy termed dendrite rearrangement that leverages the electrochemical self-discharge process to controllably address the dendrite issues. By temporarily increasing the input voltage within a single cycle, this strategy resets the cell stability without precipitating undesirable side reactions during normal operation. This pioneering technique extends operational lifespans to over three times their original duration, facilitating 80 000 cycles for Zn-ion hybrid capacitors and 3000 hours for Zn symmetrical cells. Even in scaled-up pouch cells, Ah-level symmetrical cells demonstrate a cumulative capacity nearing 200 000 mAh, significantly surpassing those of reported metal symmetrical cells. Additionally, the electrolytic Zn–MnO_2_ battery demonstrates a high capacity approaching 10 Ah, setting a new benchmark among reported ZB devices. These results mark a significant advancement towards resolving dendrite-related issues in metal anode batteries, paving the way for their sustainable development and potential commercialization.

## Teaser

A voltage-controlled dendrite rearrangement strategy is proposed to address the dendrite issues in metal anode batteries, which leverages the self-discharge process to eliminate generated dendrites, resetting cell stability and extending lifespans.

## INTRODUCTION

Recent advances in sustainable technologies, such as photovoltaics and carbon dioxide catalytic reduction, have driven unprecedented growth in the field of renewable energy [1–3]. These achievements have heightened the demand for efficient energy-storage techniques that collect and store the generated energy before its output and usage [4–6]. Rechargeable batteries with metal anodes such as lithium (Li), sodium (Na), potassium (K) and zinc (Zn) have received extensive attention due to their ultra-high specific capacity and low redox potential [7–9]. However, these battery systems are seriously hindered by rampant dendrite growth [10], which is known to not only greatly reduce the cell lifespan [11], but may also lead to other severe results, including short circuit, thermal runaway and even battery explosion [12].

The dendrite growth is derived from uneven electric field distribution, leading to induced metal deposition, which gradually deteriorates during the metal plating/striping processes and eventually results in cell short circuits [13–15]. Take aqueous zinc-based batteries (ZBs) as an example. The sources of Zn dendrite growth include surface defects formed during the production procedures of Zn foils, uneven separator channels and external pressure, etc. [16–20]. So far, various strategies have been developed to mitigate dendrite growth, such as anode coating, structural design and electrolyte regulation [21–24]. Although these approaches show promise in alleviating dendrite growth and other anode issues, they come with limitations when being applied in ZBs (Fig. S1) [25]. Surface coatings that are aimed at solving dendrite issues mainly take effect through constructing uniform ion channels with sieving properties on the anode surface to ensure an even Zn^2+^ flux. However, the coating layers may exfoliate from the anode surface after continuous Zn plating/stripping, quickly making this strategy no longer effective, especially in large-capacity scenarios. Anode structural design usually constructs 3D skeleton structures based on Zn metal or other substrate materials to increase the anode surface area to reduce the local current density and suppress the unevenly distributed electric fields. It is worth noting that the introduction of substrate materials will contribute to additional cell weight and lower energy density, while the 3D Zn structures may collapse after a few plating/stripping cycles [26]. Electrolyte regulation is known to be a simple but effective strategy, which optimizes the Zn nucleation and growth processes through surface adsorption, desolvation control and solid–electrolyte interphase formation [27,28]. On the other hand, electrolyte additives usually introduce organic components and increase cell polarization, while alternative electrolytes such as eutectic electrolytes and water-in-salt electrolytes may compromise rate performance and increase costs. Therefore, although extensive efforts have been made to solve the dendrite issues, a low-cost, highly efficient and long-term solution that does not sacrifice the cell performance at the same time is still elusive.

Herein, we propose a unique controllable dendrite rearrangement (DR) strategy that utilizes the self-discharge process to eliminate generated dendrites. By temporarily increasing the input potential in a single cycle, the DR strategy triggers the rearrangement of dendrites without introducing side reactions during normal cycles. Compared with previously proposed optimization strategies for addressing dendrite issues, this DR strategy is designed not only to suppress dendrite growth, but also to achieve dendrite rearrangement even after their formation. The working mechanism of the DR strategy and its dendrite elimination effects have been validated through multiple investigations and visualized under optical observations, while it also provides more nucleation sites for subsequent cycles. Beyond solving dendrite issues, the DR strategy also demonstrates additional effects, such as removing the passivation layers on the Zn anode and recovering the dead Zn derived from Zn exfoliation in flowing electrolyte or separator permeation. This innovative approach extends operational lifespans to over three times their original duration, facilitating 80 000 cycles for Zn-ion hybrid capacitors and 3000 hours for Zn symmetrical cells. Even in scaled-up pouch cells, Ah-level symmetrical cells demonstrate a cumulative capacity nearing 200 000 mAh, while the electrolytic Zn–MnO_2_ battery demonstrates a high capacity approaching 10 Ah. These findings offer a promising solution to address dendrite issues in metal anode batteries, paving the way for their sustainable development and commercialization.

## RESULTS AND DISCUSSION

**Figure 1.**
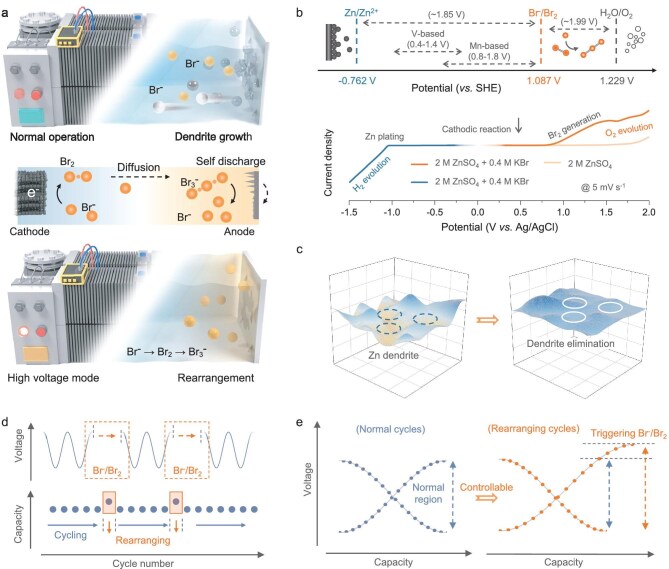
Controllable dendrite rearrangement strategy and its working mechanism elaboration. (a) Schematic illustration of the working mechanisms of the DR strategy and its application in aqueous ZBs. (b) Theoretical voltage regions for various reactions and the corresponding LSV curves. SHE stands for standard hydrogen electrode. (c) Contrast mapping images of the cycled Zn foil before and after dendrite elimination. Schematic depiction of (d) the voltage and capacity changes and (e) charge–discharge profiles during cycling and the dendrite rearrangement process.

To address the prevalent dendrite issues in metal anode batteries, the DR strategy provides a facile pathway to deal with the generated dendrites by incorporating additional rearrangement cycles that can be implemented as a specialized operational mode within practical applications (Fig. 1a). The relatively high redox potential of Br^−^/Br_2_ (>1.8 V vs. Zn/Zn^2+^) in aqueous media ensures the normal operation of cathodic reactions, such as those involving vanadium-based materials (0.4–1.4 V vs. Zn/Zn^2+^) and manganese-based materials (0.8–1.8 V vs. Zn/Zn^2+^) (Fig. 1b). Moreover, electrolyte decomposition is controlled to be excluded when the DR strategy is activated, as evidenced by the linear sweep voltammetry (LSV) curves (Fig. S2). Based on the above, the strategy is designed to eliminate the Zn dendrites that are close to the cathode, while the newborn nucleation sites on the anode will be preserved, thereby facilitating DR and sustaining its effect in subsequent cycles. During the normal cycles, dendrite growth inherently arises because of the uneven electrical field distribution. To activate the DR strategy and eliminate these dendrites, the input voltage should be raised to the Br_2_ generation region. The produced Br_2_ then diffuses through the separator channels and reacts spontaneously with the Zn dendrites. The effectiveness of the DR strategy has been confirmed in finite element simulations (FES), as illustrated in Fig. S3, demonstrating that the dendritic anode surface becomes smoother after DR. These findings are corroborated by contrast mapping images and optical microscopy observations, which document the growth and elimination of Zn dendrites (Fig. 1c and Fig. S4).

In summary, the DR strategy aims to resolve anode dendrite issues through intermittent cycles with elevated input voltages to promote sustained Br_2_ generation, diffusion and self-discharge, requiring only a marginal increase in the energy input (Fig. 1d). Consequently, the typical cell-cycling conditions based on the DR strategy are depicted in Fig. 1e, which shows the higher charge capacity and voltage in those designated single cycles.

**Figure 2.**
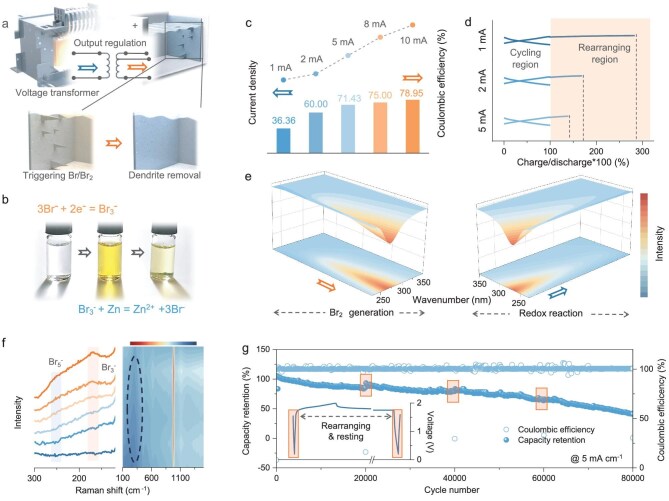
Controllable dendrite rearrangement strategy and mechanism verification. (a) Schematic illustration of regulating the input voltage to trigger the DR strategy. (b) Photograph images of the electrolyte during Br_2_ generation and the subsequent redox reaction. (c) Coulombic efficiency of single rearranging cycles under different current densities in Zn–AC hybrid capacitors and (d) the corresponding charge–discharge profiles. (e) UV spectra of the electrolyte during the Br_2_ generation and redox reaction processes. (f) Raman spectra of the electrolyte during Br_2_ generation. (g) Cycling performance of Zn–AC hybrid capacitors utilizing the DR strategy.

One key advantage of the DR strategy is its controllability: by adjusting the input voltage, Br_2_ generation can be initiated or normal cycling resumed once dendrites have been rearranged (Fig. 2a). Photographic images of the electrolyte (Fig. S5) confirm the Br_2_ generation process in the high-voltage region and the subsequent redox reaction between the Zn and Br_2_ (Fig. 2b). To further validate the working mechanism of the DR strategy, activated carbon (AC) is used as the cathode material to exclude the cathodic influences. As demonstrated by the CV curves and charge–discharge profiles in different operation modes (Fig. S6a and b), Br_2_ generation occurs when charging to a high voltage, without affecting the normal cycling of Zn–AC hybrid capacitors. It is worth noting that this strategy is also controllable by adjusting the charge current density for Br_2_ generation, as a lower current leads to greater Br_2_ generation and more severe self-discharge (Fig. 2c). In summary, the rearrangement process can be regulated by appropriately adjusting the charge current density and the upper voltage limit (Fig. 2d), for which the proper protocols should be determined based on the operational conditions.

Exploring the specific reaction pathways is a critical next step. Figure 2e presents the ultraviolet (UV) spectra of the electrolyte during the Br_2_ generation and its redox reaction with Zn particles. The peak at 270 nm corresponds to the existence of Br_3_^−^, which is formed by combining Br_2_ and Br^−^, proving that the Br_2_ diffuses in the form of polybrominated ions. The peak intensity becomes higher and then lower, reflecting the appearance and disappearance of Br_3_^−^ as Br_2_ is generated and subsequently undergoes redox reactions (Fig. S7a and b). The Raman spectra shown in Fig. 2f further confirm the presence of polybrominated ions, including Br_3_^−^ and Br_5_^−^, which gradually diminish as the redox reaction finishes (Fig. S8). Ultimately, facilitated by the periodic activation of the DR strategy in single cycles, the Zn–AC hybrid capacitors achieve an extended lifespan of 80 000 cycles and operate for >1000 hours, so they are longer-lasting compared with reported ZBs (Fig. 2g).

**Figure 3.**
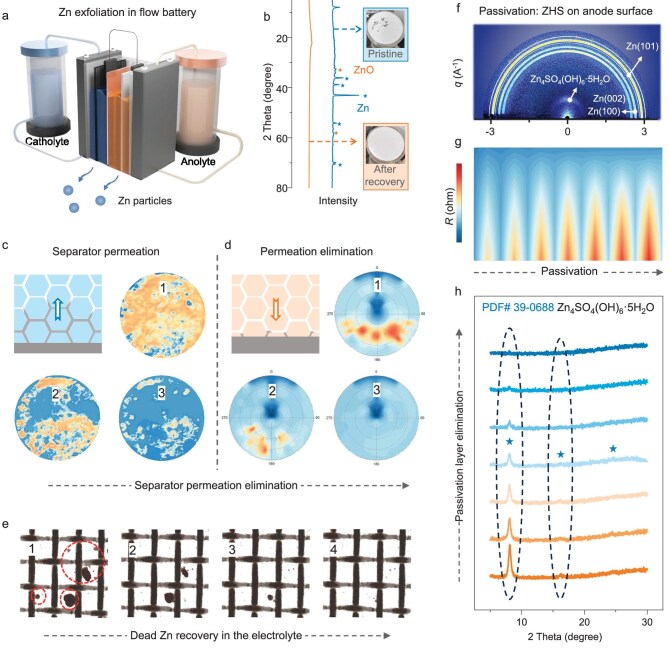
Dead Zn recovery and passivated layer elimination. (a) Schematic illustration of Zn exfoliation in a flow battery. (b) XRD spectra and the corresponding filter residues of the electrolytes, demonstrating the Zn recovery effect. Contrast figures indicating (c) the separator permeation and (d) the permeation elimination processes. (e) Optical images illustrating the recovery of dead Zn in the liquid electrolyte. (f) GIWAXS image of the passivated Zn foil. (g) Contour mapping indicating the increasing cell resistance during the ZHS formation. (h) XRD spectra indicating the elimination of the passivation layer during the rearrangement process.

In addition to dendrite elimination, the DR strategy addresses other anode issues, including dead Zn and surface passivation. Figure 3a illustrates the formation of dead Zn particles in the flowing electrolyte that exfoliate from the anode due to continuous fluid impact and weaker bonding forces. Dead Zn not only leads to anode loss, but may also obstruct the electrolyte pipeline [29]. The X-ray diffraction (XRD) spectra in Fig. 3b confirm the presence of Zn and ZnO in the filter residue, which are completely eliminated after a single rearrangement cycle. In battery systems that have separators, such as pouch and coin cells, separator permeation can cause short circuits and cell failures [30], as validated in Fig. S9 and Fig. 3c. Here, the Zn dendrites that penetrate the separators are eliminated by implementing the DR strategy, as shown in the corresponding contrast pole figures (Fig. 3d). This process is also confirmed in Fig. 3e and Fig. S10, in which intentionally added Zn particles in the electrolyte gradually disappear as Br_2_ is generated.

Passivation is another issue in nearly neutral ZBs, caused by the local increase in pH due to intrinsic anodic corrosion and hydrogen evolution. The formation of Zn_4_SO_4_(OH)_6_·5H_2_O (ZHS) on the anode surface impedes ion transport and leads to higher resistance. As demonstrated by the grazing incidence wide-angle X-ray scattering (GIWAXS) results shown in Fig. 3f, ZHS forms during battery cycling and resting, causing the cell resistance to gradually increase (Fig. 3g and Fig. S11). Thanks to the formation and diffusion of Br_3_^−^ to the anode region, the ZHS layer is first eliminated during the DR process, as evidenced by the XRD spectra (Fig. 3h and Fig. S12). Following the elimination of the ZHS layer, the Zn anode is exposed, presenting higher signals in X-ray photoelectron spectroscopy spectra (Fig. S13).

**Figure 4.**
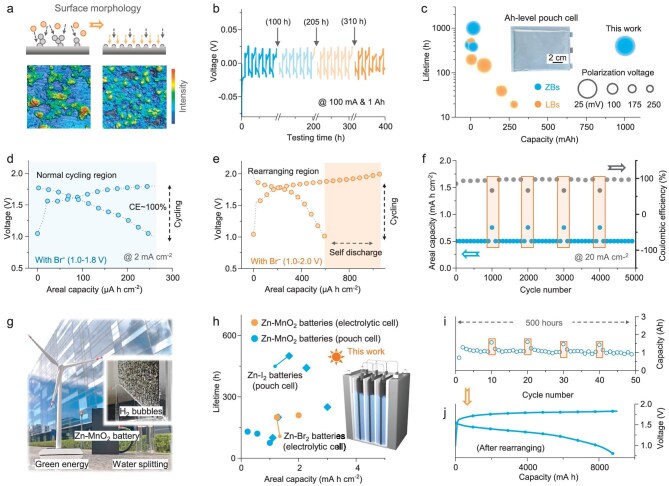
Electrochemical performance of ZBs based on the DR strategy. (a) Schematic illustration of the anode surface morphology changes after the rearrangement process, along with the corresponding CLSM images. (b) Cycling performance of the Zn symmetrical pouch cells utilizing the DR strategy. (c) Performance comparison of the symmetrical pouch cells in this work and those from other reports (Table S1). Charge–discharge profiles of (d) the normal cycles and (e) the rearranging cycles in miniature Zn–MnO_2_ batteries. (f) Cycling performance of the electrolytic Zn–MnO_2_ batteries utilizing the DR strategy. (g) Optical images of the assembled Zn–MnO_2_ battery, indicating potential applications. (h) Performance comparison of the Zn–MnO_2_ battery utilizing the DR strategy and the cells from other reports (Table S2). (i) Cycling performance of the Ah-level Zn–MnO_2_ battery and (j) the charge–discharge profile after a capacity increase.

To further investigate the unique effects of the DR strategy under long-term cycling conditions, we focused on the changes in the anode surface properties after the rearrangement processes. As shown in Fig. 4a, confocal laser scanning microscopy (CLSM) images demonstrate that the anode surface becomes smoother and the reactive area increases after DR, effectively eliminating existing dendrites and suppressing subsequent dendrite growth (Fig. S14). According to the nucleation curves shown in Fig. S15, the rearranged anode provides more nucleation sites and reduces the nucleation overpotential, particularly under higher current densities because of the higher electrolyte ionic conductivity (Fig. S16). The effect of providing more nucleation sites to suppress dendrite growth is confirmed in FES (Fig. S17), which indicates more uniform current distribution and homogeneous Zn deposition on the anode surface. Based on these results, simulation models of the pristine and rearranged anodes were constructed (Fig. S18a) and the current density distributions on the anode surface and within the electrolyte are presented in Fig. S18b. The results support that uniform current density distributions are achieved for the rearranged anode, which further suppress subsequent dendrite growth (Fig. S19).

Using 2 M ZnSO_4_ and 0.4 M KBr as the electrolyte, various tests on Zn symmetrical cells have been conducted. Rate capability tests (Fig. S20) indicate that the introduction of KBr does not trigger other side reactions (Fig. S21) but slightly lowers the polarization voltages. Long cycling tests are performed under the protocols of 5 mA cm^−2^ and 1 mAh cm^−2^, with DR processes at specific points. The results show that the symmetrical cell without rearranging fails after 1000 hours whereas another cell achieves a 3-fold lifespan, lasting for >3000 hours when the DR strategy is used (Fig. S22). Furthermore, Ah-level Zn symmetrical cells are assembled with additional rearranging electrodes (Fig. 4b) to validate the DR strategy in scaled-up pouch cells, which achieve a lifetime of 400 hours under 100 mA and a cumulative capacity nearing 200 000 mAh under 1000 mA, respectively (Fig. S23), indicating outstanding performance compared with other reported symmetrical pouch cells, including those with Zn anodes and Li anodes (Fig. 4c).

To evaluate the optimization effects of the DR strategy in full cells, MnO_2_ has been used as the coupled cathode material due to its moderate reaction voltage range. As shown in Fig. S24a, normal reactions in Zn–MnO_2_ batteries occur on the electrodes during normal cycles, respectively, while the Br^−^/Br_2_ reaction is triggered when raising the input voltage. Fig. S24b presents the potentials of cathodic reactions and their voltage regions to guide the determination of the proper input voltage. Notably, the MnO_2_ reactions, particularly MnO_2_ deposition, also occur in a higher-voltage region, potentially leading to higher discharge capacity even in rearranging cycles. Eventually, the voltage range of 1.0–1.8 V is chosen as the normal cycling region for Zn–MnO_2_ batteries (Fig. 4d), while a higher voltage limit (according to practical scenarios) is used in the single cycles to activate the DR strategy without leading to the oxygen evolution reaction (Fig. 4e). Fig. S25 presents the CV curves of the Zn–MnO_2_ batteries between 1.0 and 1.8 V, indicating that KBr addition does not induce side reactions in the normal cycling region (Fig. S26). As shown in Fig. S27, the peak currents in the CV curves of the Zn–MnO_2_ battery with KBr addition are slightly higher, which should be attributed to lower cell resistance, as mentioned above. The assembled miniature Zn–MnO_2_ batteries suffer from a short circuit after 600 cycles (Fig. S28), but the lifetime is extended to 1500 cycles by introducing single rearranging cycles (Fig. S29). Ultimately, under the cycling protocols of 20 mA cm^−2^ and 0.5 mAh cm^−2^, the electrolytic Zn–MnO_2_ batteries achieve a lifespan of 5000 cycles (Fig. 4f).

The practical applications of ZBs have faced several challenges, one of which is the intrinsic hydrogen evolution reaction (HER) that causes the assembled pouch cells to swell, leading to accelerating failures and serious consequences. We propose that open cell systems are more suitable for ZBs due to their air stability, making HER less of a concern. As shown in Fig. 4g, green energy storage is one of the potential applications of ZBs, including solar and wind energy, which can be further converted into hydrogen energy through water splitting. During these processes, anode stability is crucial for ensuring cell operation, making the DR strategy even more significant. Figure 4h presents a performance comparison between the ZBs that have been reported and the assembled Ah-level Zn–MnO_2_ battery in this work, which exhibits a lifetime of 500 hours (Fig. 4i and Fig. S30). Moreover, a high capacity approaching 10 Ah is achieved without short circuits after utilizing the DR strategy (Fig. 4j).

## CONCLUSION

In this work, we have introduced a controllable DR strategy to address the persistent issue of notorious dendrite growth in metal anode batteries. Unlike previous strategies that merely aim to suppress dendrite growth, the DR strategy actively eliminates dendrites that are generated in previous cycles and can be combined with other suppression techniques (Fig. S31). The working mechanism of the DR strategy has been validated through multiple experiments, demonstrating that the Br^−^/Br_2_ reaction occurs on the cathode within a specific voltage range, allowing polybrominated ions to diffuse to the anode region to initiate the redox reaction between Zn dendrites and Br_3_^−^. These processes are triggered by a temporary increase in input voltage and do not introduce side reactions during normal cycles. Furthermore, the DR strategy addresses other anode issues such as surface passivation, separator permeation and Zn exfoliation in flowing electrolytes, which are known to lead to dead Zn, performance degradation and cell short circuits. As a result, Zn-ion hybrid capacitors achieve a long lifetime of 80 000 cycles and Zn symmetrical cells operate for 3000 hours. Even in scaled-up pouch cells, Ah-level symmetrical cells demonstrate a cumulative capacity nearing 200 000 mAh, while the electrolytic Zn–MnO_2_ battery achieves a high capacity approaching 10 Ah. This work offers a promising avenue to address dendrite issues in metal anode batteries, potentially paving the way for their sustainable development and commercialization. By aligning future efforts in related fields such as intelligent variable voltage charging technology, the DR strategy can be better adapted to practical scenarios with optimized parameters.

## Supplementary Material

nwaf013_Supplemental_File
