# Supplementary material for: Electrochemical dendrite management via voltage-controlled rearrangement
Source: Natl Sci Rev. 2025 Jan 13;12(4):nwaf013. doi: 10.1093/nsr/nwaf013 (PMC11887854; doi:10.1093/nsr/nwaf013)
Supplement: nwaf013_Supplemental_File [file nwaf013_supplemental_file.pdf]

Supporting Information for

Electrochemical Dendrite Management via Voltage-Controlled Rearrangement

Zhexuan Liu[1], *Xinru Wu*[1], *Xiao Xiao*[1], *Zhiqiang Xiao*[1], *Qingjin Fu*[1], *Zhiyang Zheng*[1], *Xiongwei Zhong*[2], *Fengyi Zheng*[1], *Guangmin Zhou*[1]*

[1]Tsinghua Shenzhen International Graduate School, Tsinghua University, Shenzhen 518055, P. R. China.

[2]Department of Materials Science and Engineering, Southern University of Science and Technology, Shenzhen 518055, P. R. China.

*Corresponding E-mail: guangminzhou@sz.tsinghua.edu.cn

Methods

Materials: All the reagents are used as received without further purification. Zinc sulfate heptahydrate ($ZnSO_4 \cdot 7H_2O$, ≧99 %), potassium bromide (KBr, ≧98 %), Manganese sulfate hydrate ($MnSO_4 \cdot H_2O$, ≧99 %), Activated carbon (AC, 200 mesh, ≧99 %), Zn foil (≧99 %), Ti foil (≧99 %), were purchased from Aladdin.

Collecting permeated separators: Polypropylene separators are applied to clearly visualize the permeation. Generally, three layers are used in a single coin cell, which will be discharged until the short circuit occurs. Disassembling the cell, the separators can be simply taken out without adhesion with Zn foils.

Pretreatment of carbon cloth: The carbon cloth is cut into needed pieces, followed by ultrasonic washing in deionized water and ethanol. Then the dried pieces are immersed in the solution of 10 wt% H_2SO_4 and 10 wt% HNO_3 at 60°C for 2 h, before they were used as current collectors.

Finite element simulation: The phase-field model has been coupled with secondary/ triple current density distribution to investigate the local current density distribution, electric field distribution, ion concentration distribution, and Zn deposition simulations under various scenes with different current density and separators.

Material Characterizations: XRD patterns were obtained by Bruker D8 Advance at a rate of 10 ° min-1. The surface and interface morphology are captured by optical microscope (LW750LJT). X-ray photoelectron spectra (XPS) results are collected to investigate the sample valence bond information (ESCALAB 250 Xi X-ray Photoelectron spectrometer, Thermo Fisher). Finite Element Simulation results are

obtained using the COMSOL Multiphysics software.

Electrochemical measurements: The cells are assembled into common 2032-type coin cells or pouch cells as mentioned above. CV curves are carried out using an electrochemical station (CHI660E, China). The charge-discharge experiments were performed using a NEWARE (CT-4008Q-5V100mA-124, Shenzhen, China) or LAND (CT3001A) battery testing system at different current densities at room temperature. The electrolyte dosage for coin cells with liquid electrolyte is 150 μL.

Figures and Tables

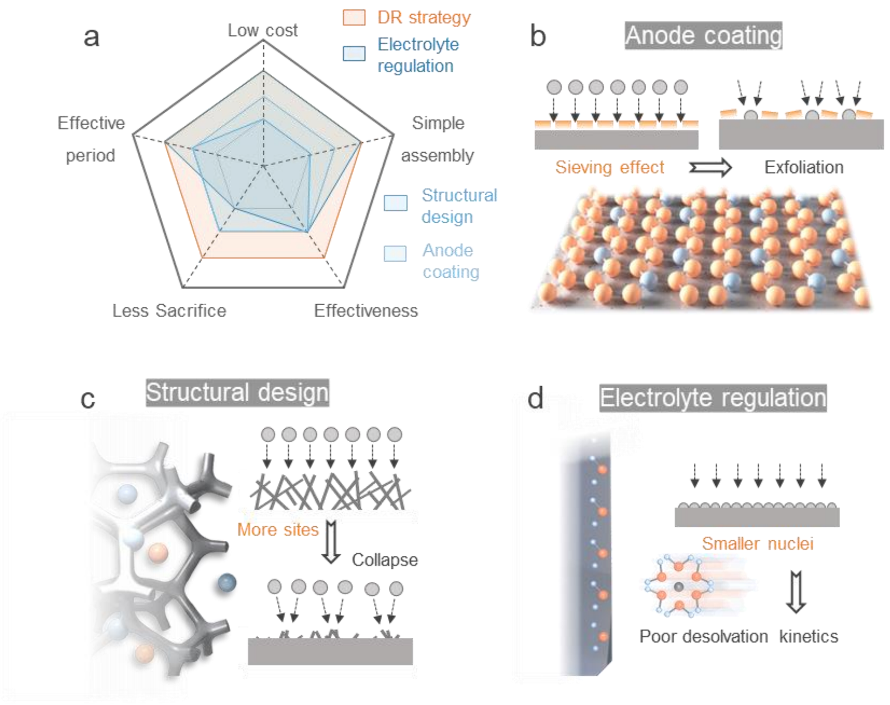

Figure S1. a) Comparation between DR strategy and the other reported strategies on properties including low cost, simple assembly, effectiveness, less sacrifice, and effective period. Schematics of the popular optimization strategies and their disadvantages: b) Anode coating, c) Structural design, and d) Electrolyte regulation.

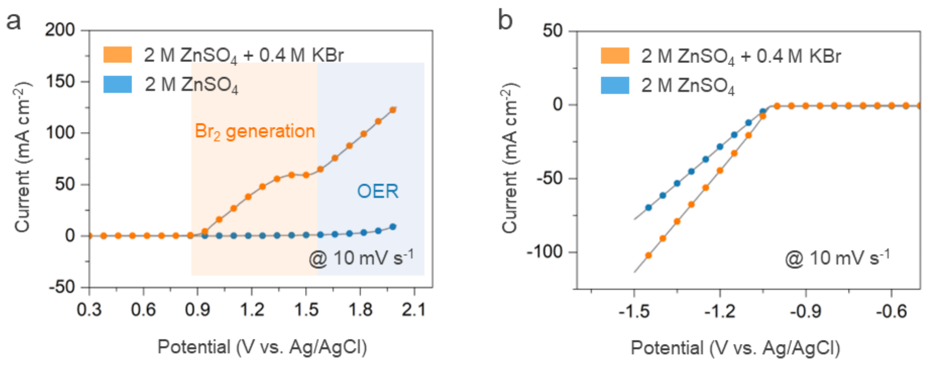

Figure S2. LSV curves in different electrolytes of the potential range between (a) 0.3 to 2.1 V and (b) 0 to -1.5 V.

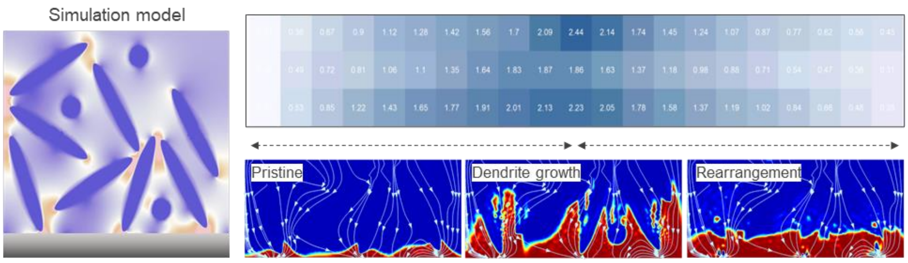

Figure S3. Simulations on Zn deposition and dendrite rearrangement.

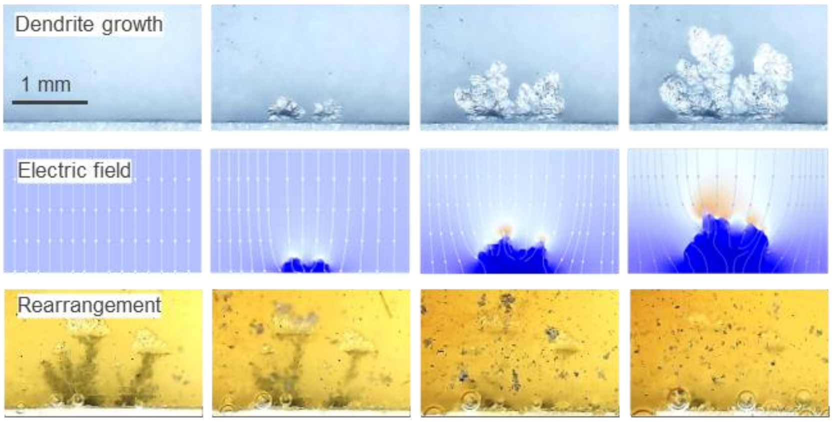

Figure S4. In-situ optical microscopy observation and the corresponding electric field simulations of the dendrite growth and rearrangement.

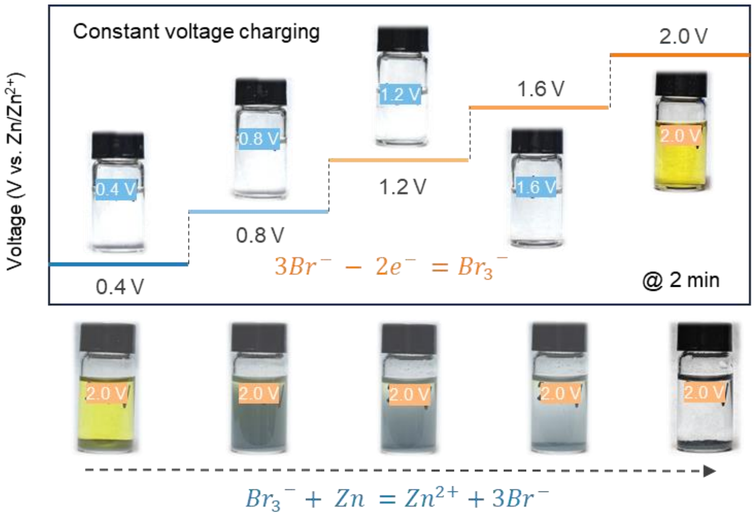

Figure S5. Optical images of the electrolyte being charged at different potentials and the redox reaction process.

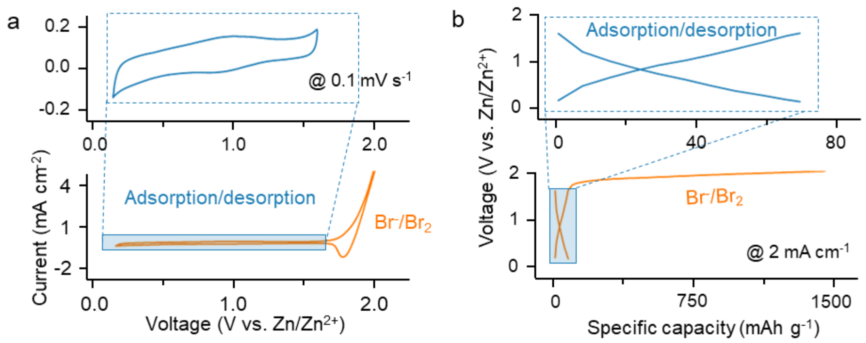

Figure S6. a) CV curves of the Zn-AC batteries under different voltage regions, and b) the corresponding charge-discharge profiles.

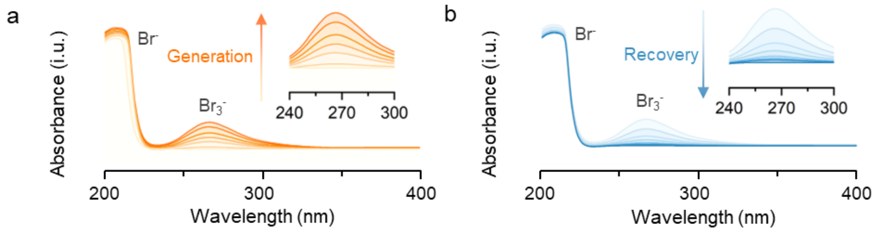

Figure S7. UV spectra of the electrolyte during a) Br_2 generation and b) redox reaction.

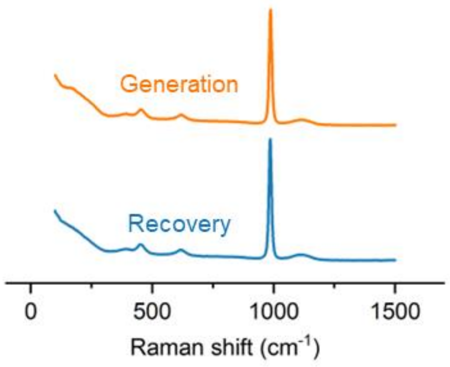

Figure S8. Raman spectra of the electrolyte after Br_2 generation and redox reaction.

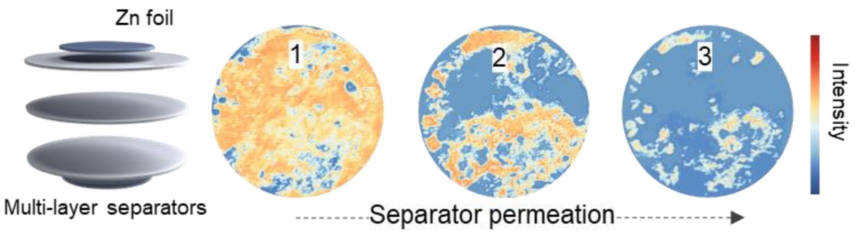

Figure S9. Schematics of the separator permeation structure and the contrast pole figures indicating its result.

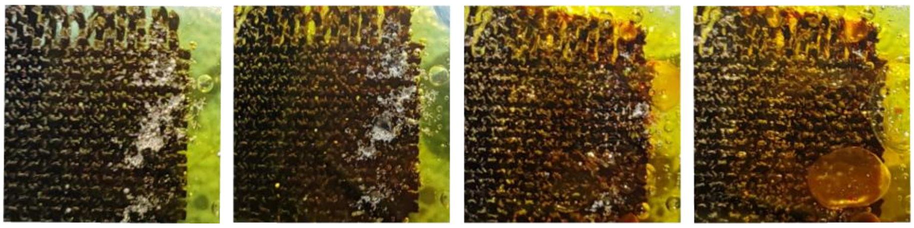

Figure S10. Optical images of the Br_2 generation on the carbon cloth cathode and the redox reaction between it and Zn powder.

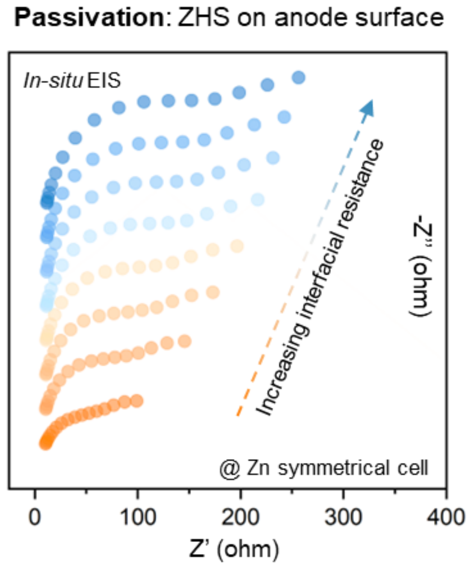

Figure S11. EIS spectra indicating the increasing interfacial resistance of the passivated anode.

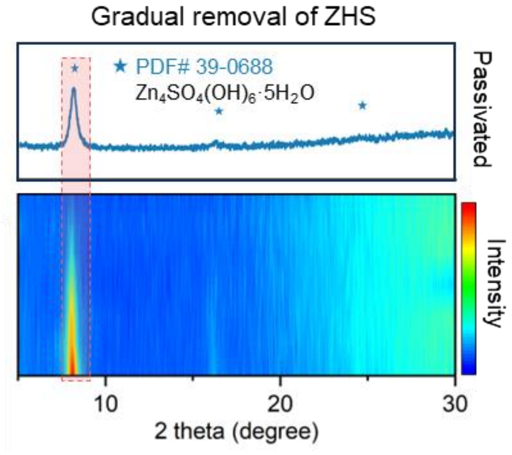

Figure S12. XRD spectra showing the gradual removal of ZHS by reacting with generated Br_2.

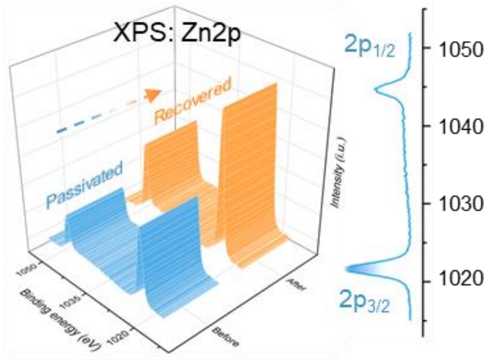

Figure S13. XPS spectra of the passivated anode and recovered anode indicating the passivation layer elimination.

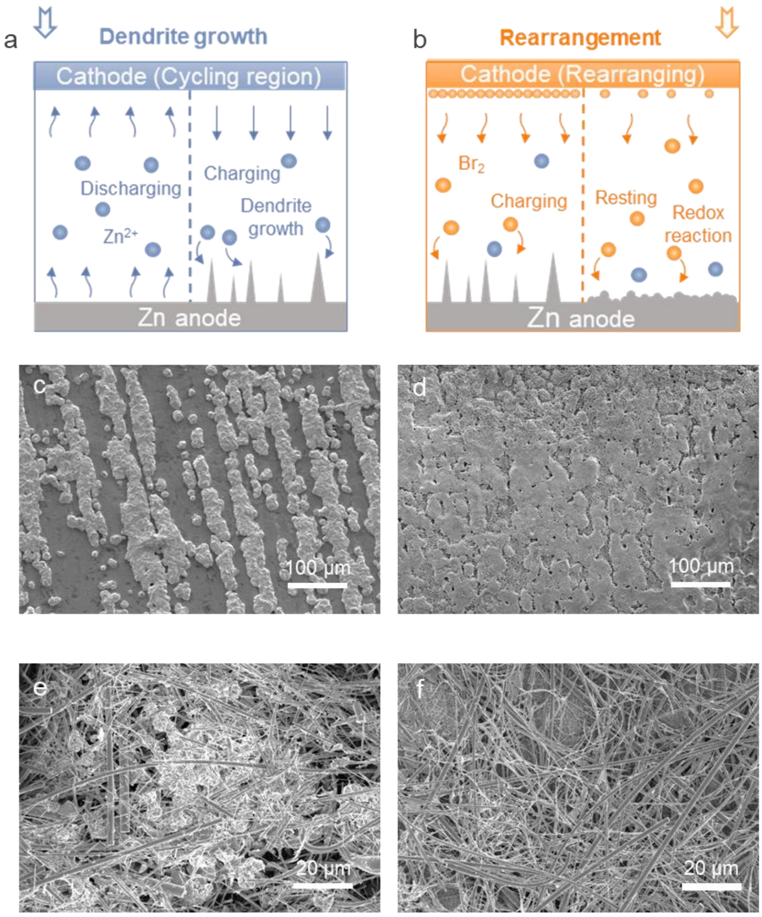

Figure S14. Schematics of a) the formation of Zn dendrites during continuous plating /stripping and b) the effects of the DR strategy. SEM images of the Zn foil c) before and d) after dendrite rearrangement, and the glassy fiber separators e) before and f) after dendrite rearrangement.

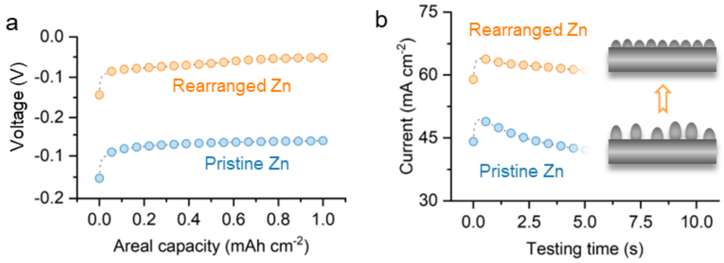

Figure S15. a) Nucleation curves and b) time-current curves of the Zn foil under different nucleation rates before and after rearranging.

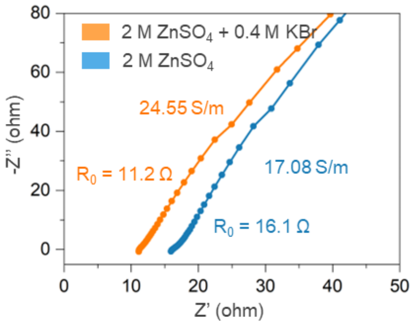

Figure S16. EIS spectra of different electrolytes with Ti foils as the inactive electrodes and the corresponding ionic conductivity.

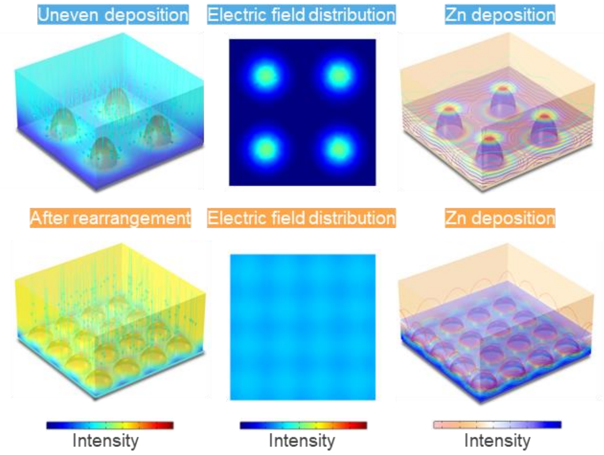

Figure S17. Simulations on the nucleation site number impacting Zn deposition.

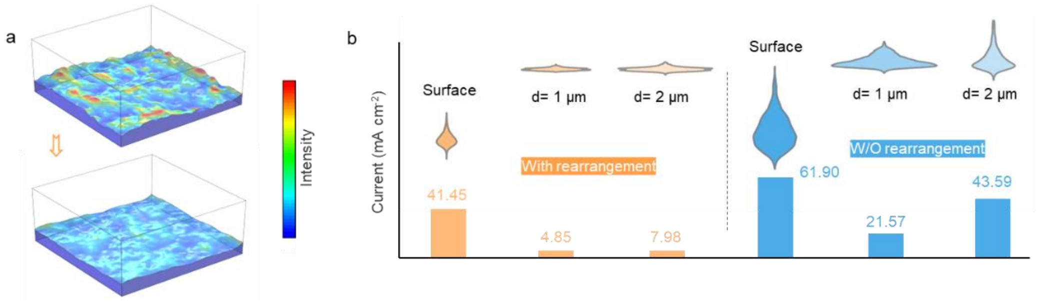

Figure S18. a) Models of the anode used in the simulations. b) Current distributions on the electrode surface and within the electrolyte.

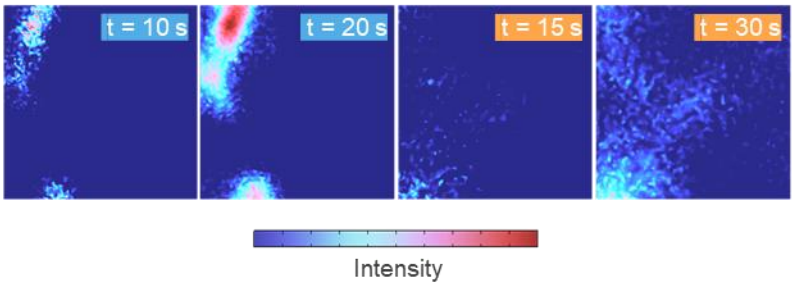

Figure S19. Simulation results of Zn deposition on pristine Zn and rearranged Zn.

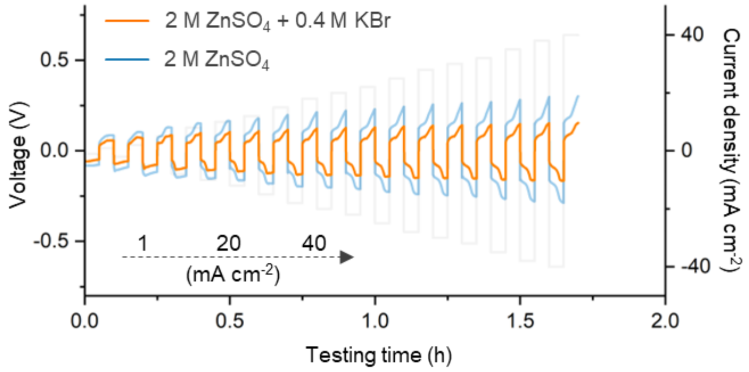

Figure S20. Rate performance of the Zn symmetrical cells in different electrolytes.

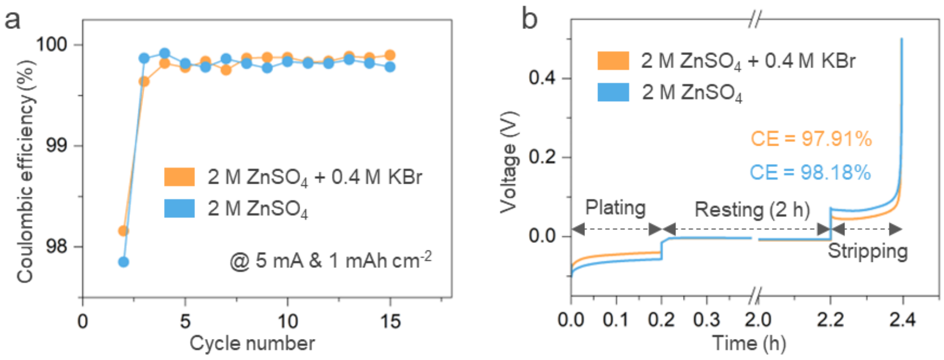

Figure S21. a) Coulombic efficiency during the initial 15 cycles of the Zn-Cu asymmetrical cells in different electrolytes. b) Coulombic efficiency of the cycle with a 2 h resting period between charge and discharge processes.

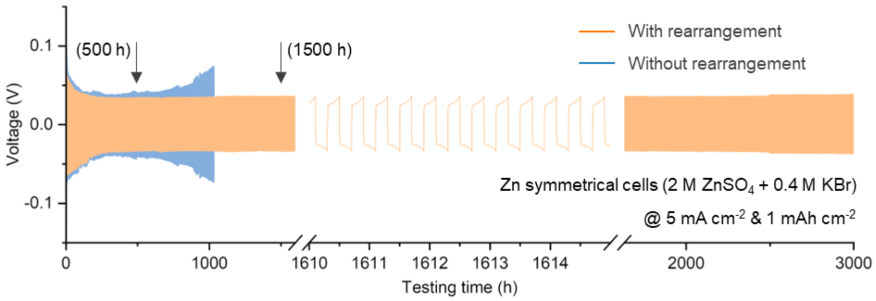

Figure S22. Rate performance of the Zn symmetrical cells in different electrolytes.

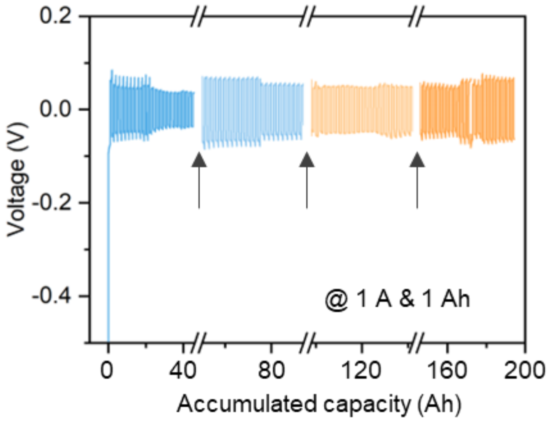

Figure S23. Cycling performance of the Zn symmetrical cells under 1 A.

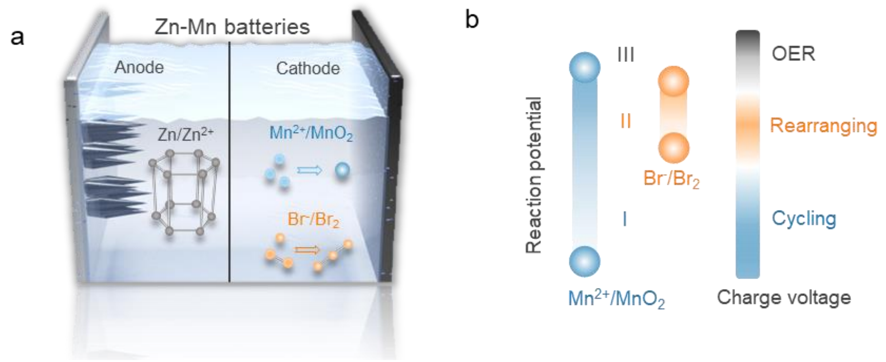

Figure S24. a) Schematic illustration of the DR strategy in Zn-MnO_2 batteries and b) the corresponding cathodic reaction regions.

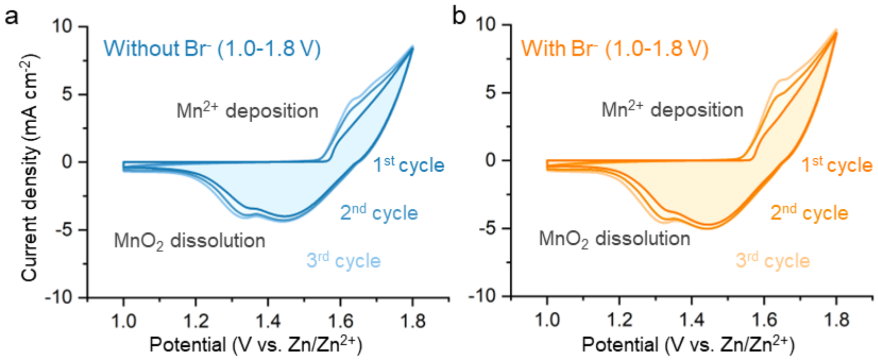

Figure S25. CV curves in the first three cycles of the Zn-MnO_2 batteries in different electrolytes.

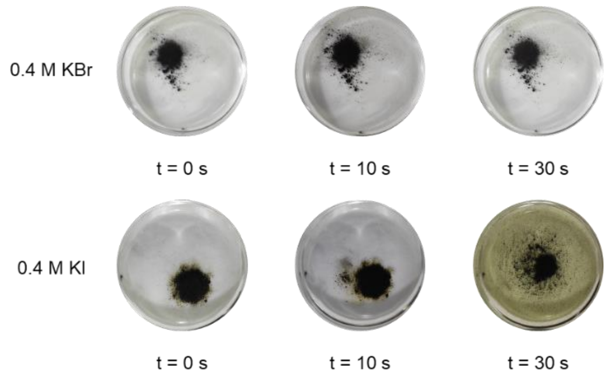

Figure S26. Optical images during the redox reaction between MnO_2 and I^-/Br^- in 2 M $ZnSO_4$ + 0.4 M KI/KBr.

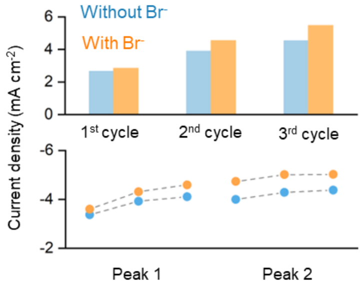

Figure S27. The peak current densities in CV curves of Zn-MnO_2 batteries with and without KBr addition.

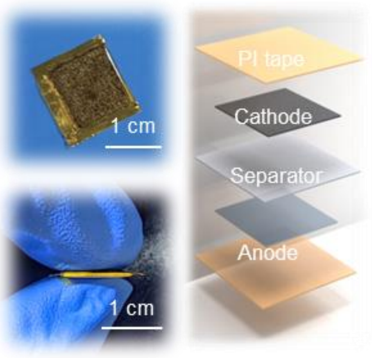

Figure S28. Schematics and optical images of the assembled miniatured Zn-MnO_2 batteries.

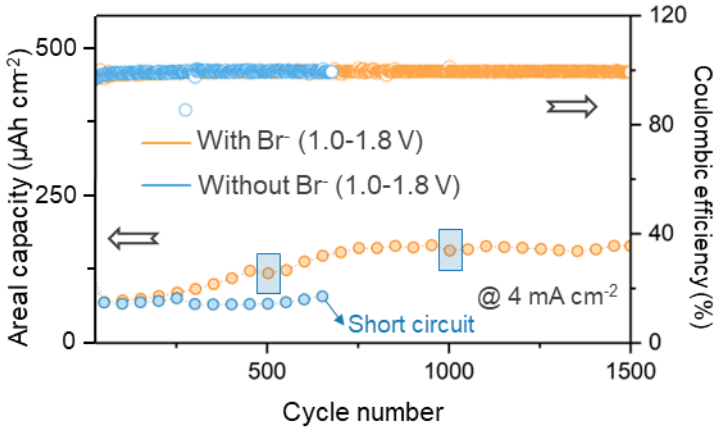

Figure S29. Cycling performance of the miniatured Zn-MnO_2 batteries.

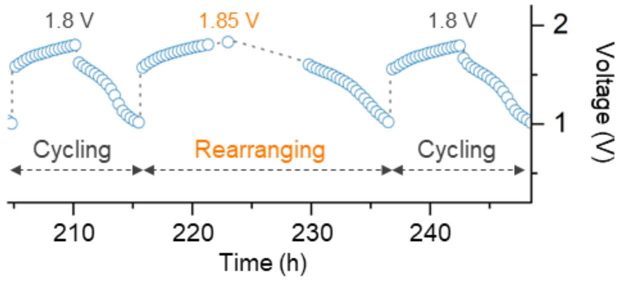

Figure S30. Charge-discharge profiles of the Ah-level Zn-MnO_2 batteries.

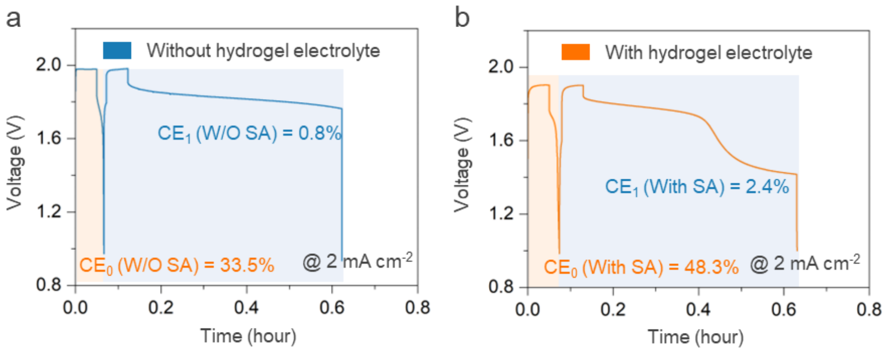

Figure S31. Charge and discharge profiles of the Zn-Br batteries with and without the hydrogel electrolyte.

Table S1. Performance comparison of symmetrical pouch cells.

Number	Capacity (mAh)	Lifetime (h)	Polarization (mV)	Ref.
1	1000	400	25	This work
2	84	150	50	
3	196	40	100	1
4	280	20	250	
5	0.375	500	200	2
6	6	400	150	3
7	2.4	200	100	4
8	9	400	100	5
9	15	1000	50	6

Table S2. Performance comparison of full cells.

Number	Areal capacity (mAh cm^{-2})	Lifetime (h)	Ref.
1	3.33	500	This work
2	2	210	7
3	0.564	120	8
4	0.227	130	9
5	1	75	10
6	1.25	200	11
7	1.1	100	12
8	3	250	13
9	1.29	200	14

Reference

1. Shi, P. *et al.* Electrochemical Diagram of an Ultrathin Lithium Metal Anode in Pouch Cells. *Adv. Mater.* **31**, 1902785 (2019).
2. Huo, H. *et al.* Evaluating Interfacial Stability in Solid-State Pouch Cells via Ultrasonic Imaging. *ACS Energy Lett.* **7**, 650-658 (2022).
3. Tao, L. *et al.* Dual-protected zinc anodes for long-life aqueous zinc ion battery with bifunctional interface constructed by zwitterionic surfactants. *Energy Storge Mater.* **63**, 102981 (2023).
4. Wei, Y. *et al.* Enabling All-Solid-State Li Metal Batteries Operated at 30 ℃ by Molecular Regulation of Polymer Electrolyte. *Adv. Energy Mater.* **13**, 2203547 (2023).
5. Zhou, M. *et al.* Intrinsic structural optimization of zinc anode with uniform second phase for stable zinc metal batteries. *Energy Storge Mater.* **52**, 161-168 (2022).
6. Zhang, J. *et al.* Nonepitaxial electrodeposition of (002)-textured Zn anode on textureless substrates for dendrite-free and hydrogen evolution-suppressed Zn batteries. *Adv. Mater.* **35**, 2300073 (2023).
7. Liu, Z. *et al.* Balanced Interfacial Ion Concentration and Migration Steric Hindrance Promoting High-Efficiency Deposition/Dissolution Battery Chemistry. *Adv. Mater.* **34**, 2204681 (2022).
8. Hu, Y. *et al.* Reconstructing interfacial manganese deposition for durable aqueous zinc–manganese batteries. *Natl. Sci. Rev.* **10**, nwad220 (2023).
9. Chao, D. *et al.* An Electrolytic Zn–MnO2 Battery for High-Voltage and Scalable Energy Storage. *Angew. Chem. Int. Ed.* **58**, 7823-7828 (2019).
10. Zhu, K. *et al.* An integrated Janus hydrogel with different hydrophilicities and gradient pore structures for high-performance zinc-ion batteries. *Energy Environ. Sci.* **17**, 4126-4136 (2024).
11. Zheng, X. *et al.* Constructing robust heterostructured interface for anode-free zinc batteries with ultrahigh capacities. *Nat. Commun.* **14**, 76 (2023).

12. Wu, J., Yang, J.-L., Zhang, B. & Fan, H. J. Immobilizing Polyiodides with Expanded Zn2+ Channels for High-Rate Practical Zinc-Iodine Battery. *Adv. Energy Mater.* **14**, 2302738 (2024).
13. Wang, K. *et al.* An Iodine-Chemisorption Binder for High-Loading and Shuttle-Free Zn–Iodine Batteries. *Adv. Energy Mater.* **14**, 2304110 (2024).
14. Kumankuma-Sarpong, J. *et al.* Entanglement Added to Cross-Linked Chains Enables Tough Gelatin-Based Hydrogel for Zn Metal Batteries. *Adv. Mater.* **36**, 2403214 (2024).
